# Melatonin in Alzheimer’s Disease

**DOI:** 10.3390/ijms140714575

**Published:** 2013-07-12

**Authors:** Li Lin, Qiong-Xia Huang, Shu-Sheng Yang, Jiang Chu, Jian-Zhi Wang, Qing Tian

**Affiliations:** 1 Key Laboratory of Neurological Disease of National Education Ministry and Hubei Province, Department of Pathology and Pathophysiology, Tongji Medical College, Huazhong University of Science and Technology, Wuhan 430030, China; E-Mail: chujiang@hust.edu.cn; 2 Department of Pathology and Pathophysiology, College of Medical Science, Jishou University, 120 People Road, Jishou 436100, China; E-Mails: linli@jsu.edu.cn (L.L.); yangshusheng@jsu.edu.cn (S.-S.Y.); 3 Department of TCM Rationale, College of Basic Medicine, Hubei University of Chinese Medicine, 1 West Road Huangjia Lake, Wuhan 430065, China; E-Mail: huangqx@hbtcm.edu.cn

**Keywords:** Alzheimer’s disease, melatonin, tau hyperphosphorylation, beta amyloid, antioxidation, cholinergic, neuroinflammation

## Abstract

Alzheimer’s disease (AD), an age-related neurodegenerative disorder with progressive cognition deficit, is characterized by extracellular senile plaques (SP) of aggregated β-amyloid (Aβ) and intracellular neurofibrillary tangles, mainly containing the hyperphosphorylated microtubule-associated protein tau. Multiple factors contribute to the etiology of AD in terms of initiation and progression. Melatonin is an endogenously produced hormone in the brain and decreases during aging and in patients with AD. Data from clinical trials indicate that melatonin supplementation improves sleep, ameliorates sundowning and slows down the progression of cognitive impairment in AD patients. Melatonin efficiently protects neuronal cells from Aβ-mediated toxicity via antioxidant and anti-amyloid properties. It not only inhibits Aβ generation, but also arrests the formation of amyloid fibrils by a structure-dependent interaction with Aβ. Our studies have demonstrated that melatonin efficiently attenuates Alzheimer-like tau hyperphosphorylation. Although the exact mechanism is still not fully understood, a direct regulatory influence of melatonin on the activities of protein kinases and protein phosphatases is proposed. Additionally, melatonin also plays a role in protecting the cholinergic system and in anti-inflammation. The aim of this review is to stimulate interest in melatonin as a potentially useful agent in the prevention and treatment of AD.

## 1. Introduction

Alzheimer’s disease (AD) is an age-associated neurodegenerative disease and characterized by progressive loss of cognition and other neurobehavioral manifestations. Pathological hallmarks of AD include extracellular senile plaques (SP), mainly consisting of β-amyloid (Aβ), and intracellular neurofibrillary tangles (NFTs), mainly composed of abnormally hyperphosphorylated tau, a microtubule-associated protein [1]. In spite of a large number of studies undertaken, the etiology of AD is largely unknown. Many mechanisms have been proposed, including genetic predispositions (e.g., expression levels and subforms of presenilins (PS) and Apolipoprotein (Apo) E), inflammatory processes associated with cytokine releasing, oxidative stress and neurotoxicity by metal ions [2–6].

Melatonin (*N*-acetyl-5-methoxytryptamine), a tryptophan metabolite and synthesized mainly in the pineal gland, has a number of physiological functions, including regulating circadian rhythms, clearing free radicals, improving immunity and generally inhibiting the oxidation of biomolecules. Decreased melatonin in serum and cerebrospinal fluid (CSF) and the loss of melatonin diurnal rhythm are observed in AD patients [7–12]. Furthermore, the level of melatonin in CSF decreases with the progression of AD neuropathology, as determined by the Braak stages [12]. Melatonin levels both in CSF and in postmortem human pineal gland are already reduced in preclinical AD subjects, who are cognitively still intact and have only the earliest signs of AD neuropathology [8,12]. A strong correlation exists between pineal content and CSF level of melatonin [8] and between CSF and plasma melatonin levels [7], suggesting that a reduced CSF melatonin level may serve as an early marker for the very first stages of AD. In mammals, melatonin exerts some of its functions through two specific high-affinity membrane receptors, melatonin receptor 1 (MT1) and melatonin receptor 2 (MT2). Decreased MT2 immunoreactivity and increased MT1 immunoreactivity have been reported in the hippocampus of AD patients [13,14]. Although the pineal gland of AD patients has molecular changes, no changes in pineal weight, calcification or total protein content have been observed [8,15]. It is also shown that β1-adrenergic receptor mRNA disappeared, and the activity and gene expression of monoamine oxidase (MAO) were upregulated in AD patients, suggesting that the dysregulation of noradrenergic innervations and the depletion of serotonin, the precursor of melatonin, might be responsible for the loss of melatonin rhythm and reduced melatonin levels in AD [16]. Melatonin supplementation has been suggested to improve circadian rhythmicity, for example, decreasing agitated behavior, confusion and “sundowning”, and to produce beneficial effects on memory in AD patients [17–21]. Therefore, melatonin supplementation, with its marked low toxicity [22–24], may be one of the possible strategies for symptomatic treatment.

In AD, Aβ is generally believed to play an important role in promoting neuronal degeneration by rendering neurons more vulnerable to age-related increases in levels of oxidative stress and impairments in cellular energy metabolism [25]. As the major microtubule-associated protein, tau promotes microtubule assembly and stabilizes microtubules. Hyperphosphorylation will obviously reduce the abilities of tau, which leads to cytoskeletal arrangement disruption [26,27]. The extent of neurofibrillary pathology, and particularly the number of cortical NFT, correlates positively with the severity of dementia [28]. As melatonin is able to improve some of the clinical symptoms of AD, and the level of melatonin decreases dramatically during AD, studies on the relationship between melatonin and AD pathology will be helpful to assess its potential in the prevention or treatment of AD. In this review, we will address the role of melatonin in tau hyperphosphorylation and Aβ toxicity. As cholinergic deficit and inflammation are involved in AD pathogenesis, the protection of melatonin on the cholinergic system and inflammation are also introduced. Each part is described, from phenomenon observation to mechanism investigation and speculation.

## 2. Melatonin in Tau Hyperphosphorylation

Hyperphosphorylated tau has been identified in more than a dozen of neurodegenerative disorders, termed tauopathies, including AD, Niemann-Pick type C disease, and so on [29–31]. Among these tau-related disorders, AD is the most common and the best-studied tauopathy. In AD brains, the level of the hyperphosphorylated tau is 3–4-fold higher than that of tau from normal adult brains [32,33]. There are 79 putative serine or threonine phosphorylation sites in the longest human brain tau isoform, and more than 30 phosphorylation sites have been identified in AD brain, including Thr39, Ser46, Thr50, Thr69, Thr153, Thr175, Thr181, Ser184, Ser185, Ser195, Ser198, Ser199, Ser202, Thr205, Ser208, Ser210, Thr212, Ser214, Thr217, Thr231, Ser235, Ser237, Ser238, Ser245, Ser258, Ser262, Ser285, Ser293, Ser305, Ser320, Ser324, Ser352, Ser356, Thr377, Ser396, Ser400, Thr403, Ser404, Ser409, Ser412, Ser413, Ser416 and Ser422 [34–39]. The spreading of tau pathology in the brain is the hallmark of AD pathogenesis, and the number of NFTs is positively correlated with the clinical cognitive deficit of AD patients [40].

Inhibition of tau hyperphosphorylation is one target in AD treatment. Thus, we systemically studied the effect of melatonin on tau hyperphosphorylation and found that melatonin efficiently attenuates tau hyperphosphorylation induced by wortmannin [41], calyculin A (CA) [42–44] and okadaic acid [45] in N2a and SH-SY5Y neuroblastoma cells. It was further demonstrated that melatonin significantly ameliorated tau hyperphosphorylation elicited by wortmannin [46], isoproterenol [47,48], CA [44] and constant light illumination [24] in rats. To elucidate the mechanisms underlying the inhibitory effect of melatonin on tau hyperphosphorylation, alterations of the activities of protein kinases and phosphatases were detected. Melatonin treatment not only inhibited wortmannin-induced glycogen synthase kinase-3 (GSK-3) activation, isoproterenol-induced protein kinase A (PKA) activation and CA-induced protein phosphatase-2A (PP-2A) inactivation, but also antagonized the oxidative stress induced by these agents [46,49,50]. These results from our studies provide evidence for the strong efficacy of melatonin in inhibiting tau hyperphosphorylation.

To explore whether a decrease of melatonin would induce the alteration of tau phosphorylation, we inhibited melatonin biosynthesis by brain injection of haloperidol, an inhibitor of 5-hydroxyindole-*O*-methyltransferase (one key enzyme in melatonin synthesis) in rats [51]. It was found that inhibition of melatonin biosynthesis not only resulted in spatial memory impairment in rats, but also induced an increase in tau phosphorylation with a concomitant decrease in PP-2A activity. Supplementation with melatonin by prior injection for one week and reinforcement during the haloperidol administration period significantly improved memory retention deficits, arrested tau hyperphosphorylation and oxidative stress and restored PP-2A activity [51]. We also used constant illumination to interrupt melatonin metabolism in rats. Concomitant with decreased serum melatonin, the constantly illuminated rats developed spatial memory deficits, tau hyperphosphorylation at multiple sites, activation of GSK-3 and PKA, as well as suppression of PP-1. Prominent oxidative damage and organelle lesions, demonstrated by increased expression of endoplasmic reticulum (ER) stress-related proteins, including immunoglobulin-binding protein (BiP)/GRP78 and CHOP/GADD153, decreased the number of rough ER and free ribosome, resulted in thinner synapses and increased superoxide dismutase and MAO, which were also observed in the light exposed rats. Simultaneous supplementation of melatonin partially arrested the behavioral and molecular impairments [24]. Although it is unclear whether diminished melatonin concentration is one causative factor in AD pathology or only a secondary process, our results strongly implicate the important role of decreased melatonin in Alzheimer-like spatial memory impairment and tau hyperphosphorylation.

Chemical agents used in our studies, including wortmannin, isoproterenol and CA, not only induced tau phosphorylation, but also initiated oxidative stress, as manifested by an elevated level of malondialdehyde and an altered activity of superoxide dismutase (SOD) [41–43]. Furthermore, melatonin is a potent direct free radical scavenger and indirect antioxidant that acts by augmenting the activity of several important antioxidative enzymes, such as SOD, glutathione peroxidase and glutathione reductase [52]. Constant illumination not only induced the decreased serum melatonin level, but also the increased levels of SOD and MAO in rats [24]. Oxidative stress is known to influence the phosphorylation state of tau [53–55]. The accumulation of misfolded and aggregated proteins in neurons of AD brain was considered to be related to oxidative stress, along with its molecular structure changes with aging [56]. As an antioxidant and free radical scavenger [57–59], melatonin prevents the overproduction of free radicals and reduces neuronal damage resulting from a variety of pathological processes [60–62]. It is therefore possible that prevention against tau phosphorylation by melatonin is partially due to its antioxidant activity.

Some studies also indicated melatonin may act as an enzyme modulator in a way that is unrelated to its antioxidant properties. Accumulating data provide evidence for the regulation by melatonin of protein kinases, including PKA [63,64], protein kinase C (PKC) [65,66], Ca^2+^/calmodulin-dependent kinase II (CaMKII) [67,68] and the mitogen-activated protein kinase (MAPK) family [69–72]. The documented correlation between melatonin and cAMP indicates that melatonin might inhibit PKA activity through the melatonin receptor coupled inhibition of adenylyl cyclase and reduction of cAMP [63,64]. Although there is no evidence of a direct relationship between melatonin and GSK-3 activity, one of our studies has found that melatonin treatment revised constant light illumination induced GSK-3 activation in the brain of rats [24].

## 3. Melatonin and Aβ Toxicity

Aβ, composing 39–43 amino acid residues derived from its precursor, amyloid precursor protein (APP), plays a pivotal role in the pathogenesis of AD [25,73]. Mature APP is processed proteolytically by distinct α-secretase or β-secretase pathways [74]. The nonamyloidogenic α-pathway involves the cleavage of APP within the Aβ sequence by α-secretase to release an N-terminal APP fragment, which, in turn, is cleaved by γ-secretase. Thus, the cleavage by γ-secretase precludes the formation of Aβ. The amyloidogenic β-secretase pathway, which results in the formation of intact Aβ peptide, is mediated by the sequential cleavage of β-secretase and γ-secretases at the *N*- and *C*-terminals of the Aβ sequence, respectively [75]. Melatonin has been found to inhibit normal levels of secretion of soluble APP (sAPP) in different cell lines by interfering with APP full maturation [76]. Additionally, administration of melatonin efficiently reduced Aβ generation and deposition both *in vivo* [77,78] and *in vitro* [76,79–81]. We have demonstrated that melatonin reduces Aβ generation in mouse neuroblastoma N2a cells harboring APP695 [80,82].

However, an *in vivo* study showed that melatonin did not affect the expression of APP holoprotein in transgenic Tg2576 mice [77]. Furthermore, despite achieving high plasma concentrations of melatonin, chronic melatonin therapy in old Tg2576 mice initiated at 14 months of age not only failed to remove existing plaques, but also failed to prevent additional Aβ deposition [83]. This result is in contrast with those of diminished Aβ in melatonin-treated wild-type mice [78] and reduced Aβ and protein nitration in melatonin-treated Tg2576 mice [77]. The initiation time of melatonin treatment might account for the difference between the studies of Matsubara *et al.* [77] and Quinn *et al.* [83], in which the same transgenic Tg2576 mouse model was used. Amyloid plaque pathology typically appears in 10–12-month old Tg2576 mice [84]. Melatonin treatment in the study of Matsubara *et al.* was started when the mice were four months old (prior to the appearance of hippocampal and cortical plaques) [77], an earlier pathological stage compared with 14 months of age in the study of Quinn *et al.* [83]. However, both studies concur in finding little evidence of the potent antioxidant effects of melatonin in the oldest mice. These findings indicate that melatonin has the ability to regulate APP metabolism and prevent Aβ pathology, but fails to exert anti-amyloid or antioxidant effects when initiated after Aβ deposition. Although consistent conclusions were achieved, none of the related studies further explain how melatonin exerts its inhibitory effect on Aβ generation. The proteolytic cleavage of APP by the α-secretase pathway is regulated by many physiological and pathological stimuli and the PKC-dependent mechanism is one of the most recognized. Stimuli, such as muscarinic and metabotrophic glutamate receptor agonists and phorbol esters, share the capacity to stimulate soluble APP secretion and inhibit Aβ formation through PKC activation [75]. The mechanism whereby PKC activity increases soluble APP secretion is still unknown, but it may involve additional kinase steps and the eventual activation of the secretases that mediated APP cleavage. Recently, the inhibitory regulation by GSK-3 inhibition on Aβ generation has been well-established [85–87]. The mechanism behind this is not clear. It was demonstrated that inhibition of GSK-3 and upregulation of c-Jun *N*-terminal kinase (JNK) result in elevated matrix metalloprotease activity and increased degradation of Aβ [88]. As phosphorylation of GSK-3 leads to its inactivation, the data suggest that activated GSK-3 may inhibit or reduce JNK activation by certain stimuli [89]. GSK-3 interacts with presenilin-1 (PS1), a cofactor for γ-secretase, implying that GSK-3 may function as a component in the γ-secretase complex [90,91]. Assuming that melatonin can influence PKC and GSK-3 activity as mentioned earlier, it is postulated that melatonin may regulate APP processing through the PKC and GSK-3 pathways. As PKC is an upstream regulator of GSK-3, GSK-3 may be one of the common signal pathways increasing Aβ generation and tau hyperphosphorylation. Regulation of the Aβ fibril formation and the important pathological property of Aβ, such as neurotoxicity and resistance to proteolytic degradation, depend on the ability of peptides to form β-sheet structures and/or amyloid fibrils [92,93].

Intervention in the Aβ aggregation process can be considered an approach to stopping or slowing the progression of AD. Melatonin can interact with Aβ40 and Aβ42 and inhibit the progressive formation of β-sheet and/or amyloid fibrils [94–96]. The anti-fibrillogenic effect of melatonin has been demonstrated by different techniques, including circular dichroism (CD) spectroscopy, electron microscopy and nuclear magnetic resonance (NMR) spectroscopy and electrospray ionization mass spectrometry (ESI-MS) [95]. Moreover, the interaction between melatonin and Aβ appears to depend on the structural characteristics of melatonin, rather than on its antioxidant properties, because it could not be reproduced by melatonin analogs or other free radical scavengers [92,94]. Evidence derived from ESI-MS proved that there was a hydrophobic interaction between Aβ and melatonin, and proteolytic investigations suggested that the interaction took place on the 29–40 residues of the Aβ segment [95]. Results from NMR spectroscopy further confirmed a residue-specific interaction between melatonin and any of the three histidine and aspartate residues of Aβ [96]. The imidazole-carboxylate salt bridges formed by the side chains of histidine (His+) and aspartate (Asp−) residues are critical to the formation of the amyloid β-sheet structures [97], and disruption of these salt bridges promotes fibril dissolution [98].

Melatonin could promote the conversion of β-sheets into random coils by disrupting the imidazole-carboxylate salt bridges and, thus, prevent Aβ fibrillogenesis and aggregation. It is therefore possible that by blocking the formation of the secondary β-sheet conformation, melatonin may not only reduce neurotoxicity, but also facilitate clearance of the peptide via increased proteolytic degradation. It has been demonstrated that melatonin directly interacts with Aβ and prevents its aggregation [99]. Aβ treatment elicits a spectrum of cellular damage, including increases in lipid peroxidation and intracellular free calcium concentration, oxidative damage to mitochondrial DNA and the emergence of apoptotic markers [100]. Mitochondria are not only the primary site of reactive oxygen species (ROS) generation, but also the primary target of attack for ROS. Melatonin was considered to stabilize the fluidity of mitochondrial inner membranes; and binding to mitochondrial membranes was revealed [101]. Oxidative stress acts synergistically with the disturbance of intracellular calcium homeostasis. The free radical-induced membrane damage induces further calcium influx, and the resultant accentuated calcium influx, in turn, will induce the generation of further free radicals. Therefore, oxidative stress plays a central role in Aβ-induced neurotoxicity and even cell death. Aside from Aβ causing oxidative stress, it has been proposed that oxidative damage could exacerbate a vicious cycle, in which amyloidogenic processing of APP would be further facilitated to generate more Aβ, which, in turn, enhances oxidative stress [102]. Accumulating data implies that melatonin efficiently protects cells against Aβ-induced oxidative damage and cell death *in vitro* [103,104] and *in vivo* [77,105–107]. In Aβ-treated cells and animals, melatonin exerts its protective activity mainly through an antioxidant effect, whereas in APP-transfected cells and transgenic animal models, the underlying mechanism is attributed to not only its antioxidant property, but also its anti-amyloid property, including inhibition of both Aβ generation and formation of β-sheets and/or amyloid fibrils.

Additionally, some findings suggest a role for perturbed melatonin signaling in the sleep disturbances that are common in AD patients [108–110]. By *in vivo* microdialysis in Tg2576 mice, it was found that the amount of brain interstitial fluid (ISF) Aβ correlated with wakefulness. Additionally, the ISF Aβ also significantly increased during acute sleep deprivation, but decreased with infusion of a dual orexin receptor antagonist. Chronic sleep restriction significantly increased Aβ plaque formation [111]. Furthermore, administration of melatonin efficiently reduced Aβ generation and deposition, both *in vivo* and *in vitro* [76–81]. Thus, melatonin may inhibit Aβ generation and loading, but the mechanism needs further investigation.

## 4. Protection of Melatonin on the Cholinergic System

Cholinergic system deficit is also an early and primary event in the pathogenesis of AD [112]. Neurons in the nucleus basalis of Meynert, a major source of cholinergic innervation of the cerebral cortex and hippocampus, undergo a profound and selective degeneration in AD brain [113–115]. The level of acetylcholine (ACh) is decreased in the early stage of AD, whereas the activities of the synthesizing enzyme, choline acetyltransferase (ChAT), and the hydrolyzing enzyme, acetylcholinesterase (AChE), do not change until the late stage of AD [116–118]. Other biological investigations of tissue from biopsy and autopsy have found a profound decrease of ChAT activity in the neocortex of AD patients, correlating positively with the severity of dementia [102]. Although the mechanism leading to the ACh deficit is still unknown, the inhibitor of AChE has been employed as a treatment and is considered the standard of care for the treatment for mild-to-moderate AD [119].

Melatonin has protective effects on the cholinergic system. A previous study has also demonstrated that melatonin partially prevented peroxynitrite-induced inhibition of choline transport and ChAT activity in several neuronal proteins from synaptosomes and, more readily, from synaptic vesicles [120]. Additionally, it is reported that four-month melatonin treatment significantly ameliorated the neuropathological, behavioral and biochemical changes in eight-month-old APP695 transgenic mice with Aβ deposition, significant learning and memory deficit and a profound reduction in ChAT activity in the frontal cortex and hippocampus [105]. Another study also showed that similar treatment with melatonin antagonized spatial memory deficit and decreased ChAT activity in ovariectomized adult rats [121]. However, in rats infused intracerebroventricularly with Aβ for 14 days, where ChAT activity was significantly reduced, melatonin was unable to restore the activity of this enzyme [122]. Melatonin showed the inhibition only on the lipopolysaccharide (LPS)-induced increase in AChE activity, whereas no changes were observed in CSF treated rats. These results are in support of the inhibitory influence of melatonin on AChE activity in streptozotocin-induced dementia [123].

Compared with placebo, the cholinesterase inhibitors, such as donepezil, tacrine, rivastigmine and galantamine, which can resolve acetylcholine deprivation, are able to stabilize or slow decline in cognition, function, behavior and global change [124]. Melatonin secretion decreases in AD, and this decrease has been postulated as responsible for the circadian disorganization, decrease in sleep efficiency and impaired cognitive function seen in those patients. Melatonin replacement has been shown effective to treat sundowning, mild cognitive impairment (MCI), an etiologically heterogeneous syndrome that precedes dementia, and other sleep wake disorders in AD patients. Besides inhibition on AChE activity, the prospects for melatonin as a treatment for AD are also based on scavenging of reactive oxygen and nitrogen species, resolving sleep disturbance, decreasing Aβ toxicity and loading [125]. There is no clinical evidence to show which is better for AD patients regarding the AChE inhibitor and melatonin. However, the combination of these two drugs may have much better effects. Recently tacrine-melatonin hybrids were designed and synthesized as new multifunctional drug candidates for AD [126,127]. The compounds show improved cholinergic and antioxidant properties, being more potent and selective inhibitors of human AChE than tacrine and capturing free radicals better than melatonin. They exhibit low toxicity and may be able to penetrate the central nervous system [126]. Direct intracerebral administration of one of these hybrids, *N*-(2-(1H-indol-3-yl)ethyl)-7-(1,2,3,4-tetrahydroacridin-9-ylamino) heptanamide, decreased Aβ-induced cell death and amyloid burden in the brain parenchyma of APP/PS1 mice. Furthermore, the reduction in Aβ pathology was accompanied by a recovery in cognitive function [127].

## 5. Role of Melatonin in Neuroinflammation of AD

A common factor in AD pathogeny is the over activation of microglia with the consequent over expression of proinflammatory cytokines [128–130]. The accumulation of Aβ in plaques, as well as Aβ oligomers may produce sequential inflammatory/oxidative events and excitotoxicity, causing neurodegeneration and cognitive impairment [131]. Furthermore, the epidemiological studies have shown that non-steroidal anti-inflammatory drug (NSAID) use decreases the incidence of AD [132]. Aβ has been shown to act as a proinflammatory agent, activating many inflammatory components, and SP, surrounded by microglia and astrocytes, coexist with cytokines and chemokines [133]. The Aβ-induced activation of microglia is thought to be one of the major sources of the inflammatory response [134]. It has been reported that melatonin attenuates kainic acid induced microglial and astroglial responses, as determined by immunohistochemical detection of isolectin-B4 and glial fibrillary acidic protein (GFAP), the specific markers for microglia and astroglia, respectively [135]. Oral melatonin administration also attenuated Aβ-induced proinflammatory cytokines, nuclear factor-*κ*B (NF-*κ*B) and nitric oxide (NO) in the rat brain [107].

It is reported that melatonin significantly reduced the proinflammatory response, decreasing by nearly 50% the Aβ-induced levels of proinflammatory cytokines, Interleukin-1-β (IL1-β), Interleukin-6 (IL6) and tumor necrosis factor-α (TNF-α), *in vivo* [107]. Furthermore, NF-*κ*B DNA binding activity was inhibited by melatonin [136,137]. More recently, it has been demonstrated that melatonin reduces NF-*κ*B-induced IL-6 in a concentration-dependent manner in Aβ-treated brain slices [138]. Melatonin administration is also reported to reduce Aβ-induced learning and memory impairment in rats, along with a significant decrease in positive glial cells expressing NF-*κ*B-induced IL-1β in addition to complement 1q (C1q) in hippocampus [139].

## 6. Conclusions

Melatonin is one of the most powerful antioxidants acting at various levels, and the level of melatonin reduces during aging and in AD patients [24,40,140–142]. Additionally, its indirect antioxidant effects and anti-amyloid effects are based on the support of appropriate circadian phasing and anti-excitotoxic actions [68,143]. Thus, it is not surprising that melatonin is protective in numerous experimental systems and has been proposed as a treatment for AD. Recent studies from APP transgenic mice have indicated that early, long-term melatonin supplementation produces anti-amyloid and antioxidant effects, but no such effect is produced when melatonin treatment is initiated after the age of amyloid formation [76,77,79,80]. Extensive clinical trials and studies with transgenic models are necessary to confirm the role of melatonin at the late pathological stage of AD. If melatonin has no effect at the late stage of AD, studies on melatonin should be limited to the prevention of AD, rather than treatment.

Adverse reactions of melatonin may occur, such as fever on the first day of melatonin treatment, hyperkinesia or complaints of restless legs, menorrhagia, pigmentation on arms and legs, headache and abdominal reactions, thrombosis and drowsiness [16,17,144,145]. Apart from these adverse reactions, early and long-term application of melatonin may at least slow down the development of AD. Besides the positive effects in experimental systems concerning antagonism of cholinergic deficit, inflammation, fibrillogenesis and tangle formation, the sleep-promoting effects and the suppression of sundowning are important results justifying the use melatonin. Although there is evidence to postulate melatonin as a useful and therapeutic tool in MCI and AD, larger double-blind multicenter studies are urgently needed to further explore and investigate the potential and usefulness of melatonin. As decreased MT2 immunoreactivity and increased MT1 immunoreactivity have been reported in the hippocampus of AD patients [13,14], specific melatonin receptor regulators and new melatonin derivatives are expected urgently.
